# Optimized bisubstrate inhibitors for the actin N-terminal acetyltransferase NAA80

**DOI:** 10.3389/fchem.2023.1202501

**Published:** 2023-06-20

**Authors:** Line M. Myklebust, Markus Baumann, Svein I. Støve, Håvard Foyn, Thomas Arnesen, Bengt Erik Haug

**Affiliations:** ^1^ Department of Biomedicine, University of Bergen, Bergen, Norway; ^2^ Department of Chemistry and Centre for Pharmacy, University of Bergen, Bergen, Norway; ^3^ Department of Biological Sciences, University of Bergen, Bergen, Norway; ^4^ Department of Surgery, Haukeland University Hospital, Bergen, Norway

**Keywords:** bisubstrate inhibitor, N-terminal acetylation, acetyltransferase, NAA80, actin, cytoskeleton, coenzyme A

## Abstract

Acetylation of protein N-termini is one of the most common protein modifications in the eukaryotic cell and is catalyzed by the N-terminal acetyltransferase family of enzymes. The N-terminal acetyltransferase NAA80 is expressed in the animal kingdom and was recently found to specifically N-terminally acetylate actin, which is the main component of the microfilament system. This unique animal cell actin processing is essential for the maintenance of cell integrity and motility. Actin is the only known substrate of NAA80, thus potent inhibitors of NAA80 could prove as important tool compounds to study the crucial roles of actin and how NAA80 regulates this by N-terminal acetylation. Herein we describe a systematic study toward optimizing the peptide part of a bisubstrate-based NAA80 inhibitor comprising of coenzyme A conjugated onto the N-terminus of a tetrapeptide amide via an acetyl linker. By testing various combinations of Asp and Glu which are found at the N-termini of β- and γ-actin, respectively, CoA-Ac-EDDI-NH_2_ was identified as the best inhibitor with an IC_50_ value of 120 nM.

## 1 Introduction

N-terminal (Nt) acetylation is a highly abundant protein modification, occurring on approximately 80% of the human proteome (Arnesen et al., 2009; Aksnes et al., 2015). The process is catalyzed by Nt acetyltransferases (NATs), transferring an acetyl group from Acetyl CoA to the amino group of the first amino acid in the protein sequence. To date seven NATs have been found in human cells, NatA-NatF, and NatH, having distinctive features in terms of subunit composition, subcellular localization and substrate specificity (Aksnes et al., 2019). Some NATs, NatA, NatB and NatC, have broad substrate pools and act co-translationally. Other NATs have a very specific substrate pool, such as NatD co-translationally acetylating histones H2A and H4 (Songkyu et al., 2003; Hole et al., 2011), NatF post-translationally acetylating transmembrane proteins (Van Damme et al., 2011; Aksnes et al., 2015), or NatH/NAA80 post-translationally acetylating actins (Drazic et al., 2018).

**FIGURE 1:**
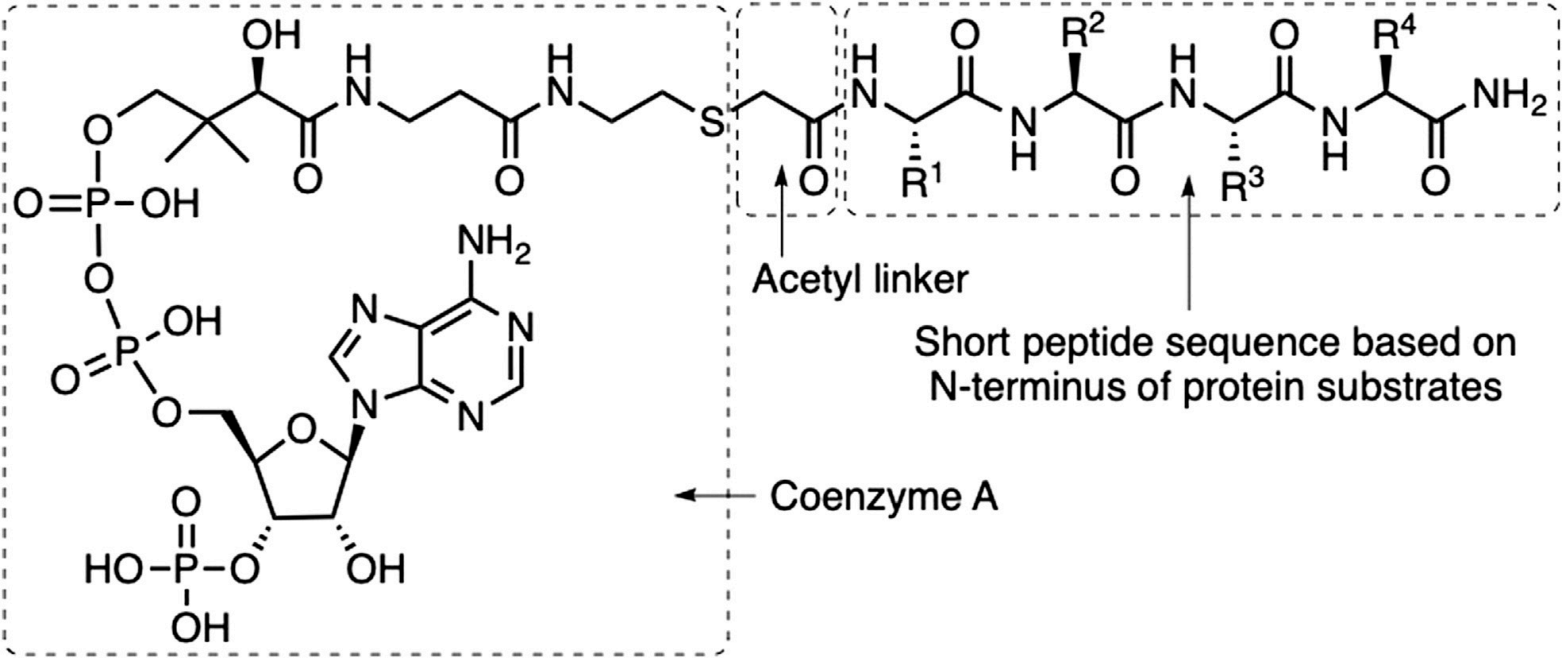
Structure of bisubstrate inhibitors. The bisubstrate inhibitors are constructed as conjugates of Coenzyme A coupled via an acetamide linker to a tetrapeptide amide. The amino acids in the peptide sequence were exchanged systematically to identify improved NAA80 inhibition.

The first catalytic mechanism of a human NAT (Evjenth et al., 2012), as well as the first structures of a NAT and NAT-complex bound to its peptide substrate, have been presented (Liszczak et al., 2011; Liszczak et al., 2013; Støve et al., 2016). Taking advantage of this information, we and others have developed peptidic NAT inhibitors that are based on covalently linking mimics of the two substrates of the biochemical reaction, i.e., coenzyme A (CoA) and a short peptide carrying an N-terminal bromoacetyl group (Figure 1) giving a thioether-linked acetyl moiety (Foyn et al., 2013; Støve et al., 2016; Goris et al., 2018; Deng et al., 2021). In these studies it has been found that it is sufficient to use the four amino acids from the N-terminus of the protein substrates to obtain potent bisubstrate inhibitors.

Such bisubstrate inhibitors demonstrate specificity and significant inhibitory potential with IC_50_ and K_i_ in the low μM/nM range. For NatD, increasing the length of the acetyl linker to propionyl has proven beneficial for the design of highly potent inhibitors (Deng et al., 2021). Further, for most NATs, access to bisubstrate inhibitors has led to significant advances in the field by enabling the determination of protein crystal structures (Liszczak et al., 2013; Støve et al., 2016; Hong et al., 2017; Goris et al., 2018; Deng et al., 2020; Deng et al., 2023).

Actin is a major component of the cytoskeleton and cytoskeletal dynamics are important for several cellular activities such as cell motility, division, and intracellular trafficking (Pollard and Cooper, 2009). The cellular activity of actin is connected to its dynamic transition between monomeric (G-actin) and filamentous (F-actin) forms. This is regulated in cells by actin-binding proteins as well as by post-translational modifications. It was established 4 decades ago that animal actins undergo a unique Nt-maturation process (Redman and Rubenstein, 1981; Rubenstein and Martin, 1983). Now we know that this involves the following steps (Arnesen and Aksnes, 2023): a) a general Nt-processing step for all eukaryotic proteins of co-translational N-terminal acetylation (Aksnes et al., 2019) (preceded by methionine excision for muscle actins), b) a unique animal actin-specific post-translational cleavage of the Nt-acetylated residue by ACTMAP (Haahr et al., 2022) and finally c) an animal actin-specific post-translational Nt-acetylation by NAA80/NatH to generate acidic actin Nt-termini (Drazic et al., 2018; Goris et al., 2018; Wiame et al., 2018). NAA80 binding to profilin, specifically PFN2, primes the interaction with actin monomers and the acetylation of actin’s N-terminus which sticks out from the folded actin monomer (Rebowski et al., 2020; Ree et al., 2020). In human cells lacking NAA80, actin is not Nt-acetylated (near 0% Nt-acetylation) while in the presence of NAA80, actin is acetylated to a near 100% stoichiometry (Drazic et al., 2018; Drazic et al., 2022). This suggests that actin Nt-acetylation is essential for optimal actin functionality in humans. Indeed, human NAA80-KO cells where actin is unacetylated display fragmentation of the Golgi apparatus, altered cytoskeletal organization including decreased G-actin/F-actin ratio, increased F-actin, increased cell size, increased filopodia and lamellopodia and increased cell migration (Aksnes et al., 2018; Drazic et al., 2018; Beigl et al., 2020). The physiological impact of actin Nt-acetylation is not fully understood due to the lack of NAA80 KO animal models. However, two brothers carrying a homozygous *NAA80* variant resulting in a partial reduction in cellular actin Nt-acetylation were recently presented (Muffels et al., 2021). These individuals showed hearing loss, mild muscle weakness and developmental delay.

Bisubstrate inhibitors of NAT enzymes could potentially be used as tools to study the effects of inhibiting Nt-acetylation to shed more light on the roles of Nt-acetylation in the cell. NAA80 inhibitors could specifically be applied to manipulate cytoskeletal dynamics and to increase cell migration. Herein we describe the optimization of a bisubstrate inhibitor for NAA80.

## 2 Material and methods

### 2.1 Synthesis of bisubstrate inhibitors

Bisubstrate inhibitors were synthesised manually or on an Initiator + Alstra (Biotage) automated microwave peptide synthesizer using Fmoc-based solid phase peptide synthesis and ChemMatrix Rink Amide resin (0.44 mmol/g loading) on a 0.25 mmol scale. 2-(6-Chloro-1*H*-benzotriazole-1-yl)-1,1,3,3-tetramethylaminium hexafluorophosphate (HCTU) and *N*,*N*-diisopropylethylamine (DIPEA) were used to couple each amino acid (3–5 equivalents) in dimethylformamide (DMF). When the automated microwave peptide synthesizer was used, the coupling process took place with microwave heating at 75 C for 5 min, while manual coupling was performed at room temperature for 30 min. The peptidyl-resin was treated with 20% piperidine in DMF for removal of the Fmoc-protecting group at room temperature for 3 + 10 min. After Fmoc-deprotection of the N-terminal amino acid, the peptidyl-resin was treated with bromoacetic acid (8 eq.) and *N*,*N′*-diisopropylcarbodiimide (DIC) in DMF for 1 h. The resin was then treated with a mixture of trifluoroacetic acid, triisopropylsilane and water (95:2.5:2.5, v/v/v) for 2 h. The suspension was filtered, and the filtrate was concentrated under reduced pressure until ∼5 mL of the solution remained. The crude product was precipitated by adding cold diethyl ether and after removal of the ether layer, the precipitate was triturated with fresh diethyl ether twice. The crude bromoacetyl peptide was dried under vacuum, purified by semi-preparative RP-HPLC and lyophilised. Purified bromoacetyl peptides and coenzyme A trilithium salt (2 eq.) were dissolved in triethylammonium bicarbonate buffer (1 M, pH 8.5) and left at room temperature overnight. Purification by RP-HPLC and lyophilisation gave the desired CoA-Ac peptides as colorless powders.

### 2.2 Recombinant protein expression and purification of HsNAA80

HsNAA80/nNat6 (NCBI gene ID:24,142) was subcloned as described by Drazic *et al.* (Drazic et al., 2018) into pETM41 vector. HsNAA80 fused to the maltose binding protein (MBP), was expressed in *Escherichia coli* BL21 star cells at 20°C overnight and lysed with sonication in lysis buffer (300 mM NaCl, 50 mM TrisHCl (pH 8.5), 1 mM DTT, 1x EDTA-free protease mixture). The recombinantly expressed MBP-hsNAA80 was further purified as described by Goris *et al.* (Goris et al., 2018)

### 2.3 *In vitro* acetyltransferase activity assay

The enzymatic activity of MBP-hNAA80 was measured using a 5,5′-dithiobis-(2-nitrobenzoic acid, DTNB) assay as described previously by Foyn *et al.* (Foyn et al., 2017) and Drazic *et al.* (Drazic et al., 2018) Briefly, the thiol group exposed in the enzymatic product CoA cleaves DTNB and produces 2-nitro-5-thiobenzonate (TNB^−^) which ionize to TNB^2-^ in neutral or alkaline pH and is readily quantified by measuring the absorbance at 412 nm.

#### 2.3.1 Nt-acetyltransferase inhibitor assays

An *in vitro* DTNB assay with enzyme (10–600 nM), 300 μM Ac-CoA, 300 μM substrate peptide, acetylation buffer (Tris, pH 8.5), and at least nine different inhibitor concentrations ranging from 0 to 500 μM was used to calculate the IC_50_ value for each bisubstrate inhibitor applied in the assay. The reaction was carried out as previously described (Drazic et al., 2018; Goris et al., 2018), and within a timeframe of 15–50 min. All measurements were performed in triplicates.

IC_50_ values were determined using GraFit 7 software and the results are summarized in Table 2.

## 3 Results and discussion

The NATs constitute an important class of enzymes and potent inhibitors can prove to be useful as tool compounds to study their roles both *in vitro* and *in vivo*. To identify such a tool compound for NAA80, we have in this work optimized the earlier identified bisubstrate inhibitor CoA-Ac-DDDI-NH_2_ (see Figure 1 for general structure). In our earlier work we tested the *in vitro* activity of NAA80 toward a broad library of potential substrates such as amino acids, nucleosides, coenzymes, various amines (e.g., serotonin and spermidine), vitamins and a number of 24-mer peptides representing the N-terminal part of proteins (Goris et al., 2018). In these substartes, the four N-terminal amino acids were varied while the remaining peptide sequence was kept constant. We found that for all of these potential substrates, NAA80 only acetylated three peptides, MDEL_24_, DDDI_24_ and EEEI_24._ DDDI_24_ and EEEI_24_ correspond to the N-terminal part of β- and γ-actin in their cytosolic processed forms, respectively, while MDEL_24_ represents the unprocessed N-terminal part of the protein p65. The level of product formation for actylation of MDEL_24_, DDDI_24_ and EEEI_24_ was determined to be 119 ± 5.61 μM, 50.5 ± 1.0 μM, and 42.4 ± 0.95 μM, respectively (Goris et al., 2018). In our inhibitor design, we decided to continue using a tetrapeptide for the protein-mimicking part as previous studies have shown that these are the most important residues for inhibitory activity (Liszczak et al., 2011; Foyn et al., 2013; Liszczak et al., 2013; Støve et al., 2016; Hong et al., 2017; Goris et al., 2018; Deng et al., 2020; Deng et al., 2021; Deng et al., 2023). Co-crystal structures of NAT-bisubstrate inhibitor complexes typically show that these four amino acids are most important for protein-inhibitor binding interactions. All inhibitors were prepared with the C-terminal carboxylic acid capped as amides to avoid the negative charge of a C-terminal carboxylic acid and better mimic a protein N-terminal.

**SCHEME 1:**
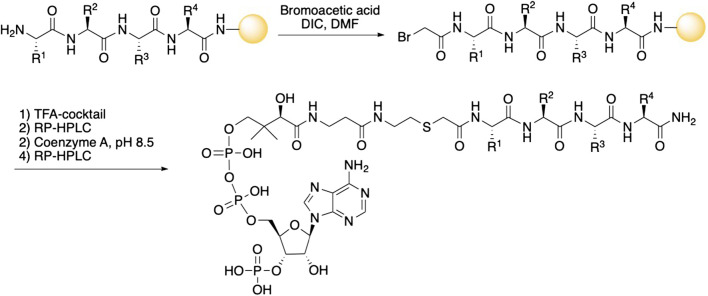
Synthesis of bisubstrate inhibitors. The peptide sequence was assembled using solid-phase Fmoc-based peptide synthesis with a bromoacetyl group at the N-terminal. Cleavage from the solid support and conjugation with Coenzyme A followed by purification gave the bisubstrate inhibitors.

The peptide part of all inhibitors was prepared using Fmoc-based solid phase peptide synthesis where the C-terminal amino acid was loaded onto a Rink amide resin (Scheme 1). After removal of the Fmoc-group of the N-terminal amino acid, coupling with bromoacetic acid introduced the acetyl-linker. The bromoacetyl peptide was then deprotected and cleaved from the resin and purified by RP-HPLC. Next, triethylammonium bicarbonate buffer (pH 8.5) was used to facilitate the conjugation of CoA to the bromoacetyl peptide. The final bisubstrate inhibitors were purified by RP-HPLC and characterized by MS and NMR to confirm their structures (see Table 1; Supplementary Table S1).

**TABLE 1:** Analytical data for bisubstrate inhibitors.

Inhibitors	Purity (%)^a^	MS obs. [M−2H]^2−^	MS calcd. [M−2H]^2−^	MS obs. [M−H]^−^	MS calcd. [M−H]^−^
CoA-Ac-DDEI-NH_2_	98.4	647.3	647.2	1,295.4	1,295.3
CoA-Ac-DEEL-NH_2_	97.9	654.3	654.2	1,309.3	1,309.3
CoA-Ac-DEEI-NH_2_	95.5	654.3	654.2	1,309.4	1,309.3
CoA-Ac-EEEL-NH_2_	94.1	661.3	661.2	1,323.4	1,323.3
CoA-Ac-EDEI-NH_2_	95.2	654.3	654.2	1,309.3	1,309.3
CoA-Ac-EDEL-NH_2_	94.7	654.3	654.2	1,309.4	1,309.3
CoA-Ac-EEDI-NH_2_	95.1	654.3	654.2	1,309.4	1,309.3
CoA-Ac-EEDL-NH_2_	97.0	654.3	654.2	1,309.4	1,309.3
CoA-Ac-EDDI-NH_2_	93.5	647.3	647.2	1,295.3	1,295.3
CoA-Ac-ESEL-NH_2_	96.8	640.4	640.2	1,281.6	1,281.3
CoA-Ac-EDQL-NH_2_	100	653.8	653.7	1,308.3	1,308.3
CoA-Ac-PDEL-NH_2_	97.3	638.3	638.2	1,277.3	1,277.3

^a^ Based on RP-HPLC analysis with monitoring at 220 nm

We have already reported that bisubstrate inhibitors based on the N-terminus of processed β- and γ-actin (DDDI and EEEI) display IC_50_ values of 0.38 and 1.16 μM, respectively (Table 2). The difference in inhibitory potency was somewhat higher than was expected based on the small difference in how well NAA80 acetylated the 24-mer peptides DDDI_24_ and EEEI_24_ as their four N-terminal amino acids. Surprisingly, while the MDEL_24_ peptide showed a higher degree of acetylation than the DDDI_24_ and EEEI_24_ peptides when we screened for potential substrates, we found that the inhibitor based on the MDEL sequence was less potent and displayed an IC_50_ value of 1.26 μM.

**TABLE 2:** Inhibitory activity.

Inhibitor	IC_50_ (μM)^a^	
CoA-DDDI-NH_2_ ^b^	0.38 ± 0.10	
CoA-EEEI-NH_2_ ^b^	1.16 ± 0.11	
CoA-MDEL-NH_2_ ^b^	1.26 ± 0.10	
CoA-DEEI-NH_2_	1.22 ± 0.33	
CoA-EDEI-NH_2_	0.76 ± 0.18	
CoA-EEDI-NH_2_	0.17 ± 0.04	
CoA-DEEL-NH_2_	4.73 ± 1.43	
CoA-EDEL-NH_2_	0.15 ± 0.02	
CoA-EDQL-NH_2_	0.85 ± 0.05	
CoA-ESEL-NH_2_	11.5 ± 3.23	
CoA-PDEL-NH_2_	13.4 ± 2.24	
CoA-EEDL-NH_2_	1.76 ± 0.52	
CoA-EEEL-NH_2_	1.16 ± 0.12	
CoA-DDEI-NH_2_	2.67 ± 0.67	
CoA-EDDI-NH_2_	0.12 ± 0.05	

^a^ All measurements were performed in triplicates. IC_50_ values are given with standard deviation.

^b^ Data taken from Goris *et al.* (Goris et al., 2018)

**FIGURE 2:**
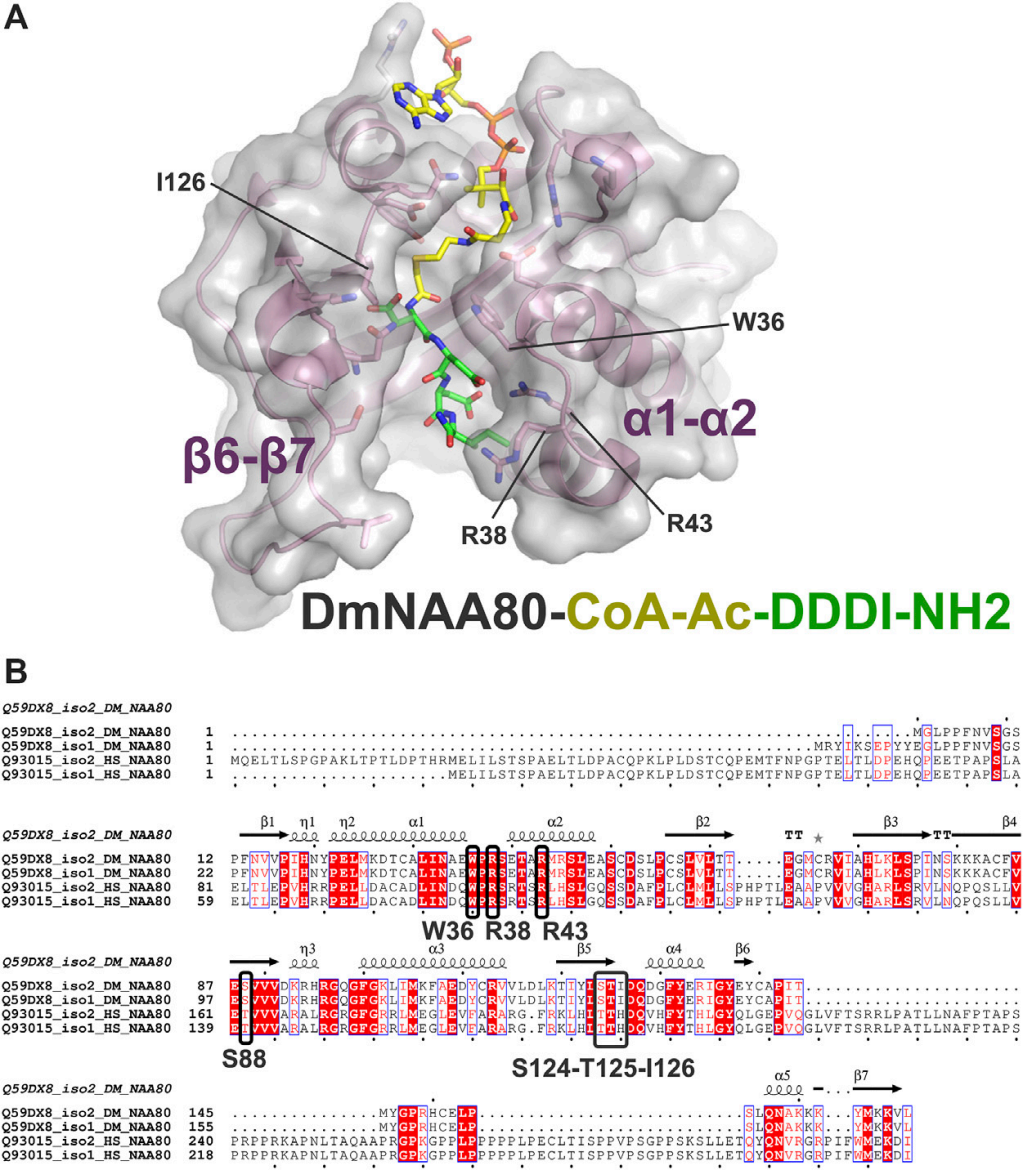
Structure of the binary *Drosophila melanogaster* (DM)NAA80/CoA-Ac-DDDI-NH_4_ complex and similarities between *Drosophila* and human NAA80 protein sequences. **(A)** The structure of DmNaa80 is represented with transparent grey surface and pink secondary structures. Side chains involved in binding of the peptide is indicated with sticks, while the bisubstrate inhibitor is highlighted as colored sticks. Key regions (α1- α2, β6-β7) and amino acids (W36, R38, R43, and I126) involved in the peptide binding of the inhibitor are labelled. **(B)** Sequence alignment of DmNAA80 and *Homo sapiens* (Hs) NAA80. The blue boxes represent sequence conservation while highly conserved residues are shown in red. Strickly conserved residues are white on a red background. Important residues directly involved in peptide inhibitor binding are marked in bold black and with black boxes. Secondary structures and sequence numbering are shown above for the DmNAA80 iso2 sequence. The alignment was made using Clustal T-Coffee and ESPript 3.0.

To elucidate inhibitor binding we have previously solved the crystal structure of DmNAA80 bound to a DDDI bisubstrate inhibitor (Goris et al., 2018). This structure revealed that mainly the α1-α2 region of NAA80 contributed to peptide binding in the inhibitor, while the β6-β7 loop which is important for peptide binding in many of the other NATs, is slightly shifted away from the peptide without contributing to inhibitor binding (Figure 2A). More specifically, D1 of the inhibitor was stabilised by a hydrogen bond from the backbone oxygen of D1 to the backbone nitrogen in S88, while the D1 side chain was stabilized by hydrogen bonds to the side chain of S124, and through a water-mediated hydrogen bond to T125 and I126. D2 of the inhibitor formed a hydrogen bond with W36 and a salt bridge with R43. Further, D3 made a water-mediated contact with R43 and hydrogen bonds with S46 and S48 and finally, the backbone oxygen of I4 formed a hydrogen bonds with R38. As can be seen from the multiple sequence alignments of DmNAA80 and hNAA80 (Figure 2B), most of the key contributing residues to binding in the DmNAA80-DDDI structure and hNAA80-profilin-actin ternary complex (W36, R43, R38 in DmNAA80 iso2 (Goris et al., 2018) corresponding to W105, R107 and R112 in hNAA80 iso 2 (Rebowski et al., 2020) is conserved. However, a key difference between the two is the extended region around the β6-β7 loop in hNAA80, a region that has shown importance for substrate binding in other NATs (Liszczak et al., 2011; Liszczak et al., 2013; Støve et al., 2016; Hong et al., 2017; Goris et al., 2018), and likely further contributes to optimimal binding of the peptide part of the bisubstrate analogue and increased potency of these inhibitors.

Since the DDDI-inhibitor was found to be significantly more potent that the EEEI-inhibitor, we decided to investigate whether the introduction of Asp into the EEEI sequence would increase inhibitory activity. It turned out that the introduction of Asp in position three was the most beneficial and the inhibitor based on the EEDI sequence showed almost ten-fold higher inhibitor activity compared to EEEI (Table 2). The EEDI sequence also proved to outperform DDDI and was found to inhibit NAA80 with an IC_50_ of 0.17 μM. Inhibitors based on the DEEI and EDEI sequences gave comparable or slightly improved IC_50_ values respectively compared to the EEEI inhibitor.

We also tested replacing the Ile residue with a Leu and found that the EEEI and EEEL sequences gave inhibitors with equal potency. On the other hand, the DEEL and EEDL sequences gave however a loss of activity compared to DEEI and EEDI while the inhibitor based on EDEL showed a four-to five-fold increase in potency compared to EDEI. Replacing Glu in position three of the EDEL sequence with a Gln residue led to an increase in IC_50_ from 0.15 μM to 0.85 μM and the replacement of the Asp residue with Ser led to a dramatic loss of potency. Interestingly, a PDEL sequence proved to give only slightly lower inhibitory potency compared to the ESEL sequence.

The introduction of two Asp residues into the EEEI sequence gave inhibitors with a large difference in inhibitory potency as the DDEI sequence gave an IC_50_ of 2.67 μM, where CoA-Ac-EDDI-NH_2_ turned out to be the most potent inhibitor with an IC_50_ of 0.12 μM.

The NAA80/NatH is a particular case among the NAT enzymes in having actin as its sole substrate. The various cytoplasmic and muscle actin substrates all have different combinations of Asp and Glu in the first N-terminal positions (Aksnes et al., 2019). Here, we show that using specific combinations of Asp and Glu in the peptide sequence makes us able to identify a highly potent NAA80 inhibitor. In conclusion, we have found that for NAA80, the choice of acidic residue at different positions of the peptide part of bisubstrate inhibitors is important and varying these has led to an optimized inhibitor with up to 3 fold higher inhibitory activity compared to those previously described. The CoA-Ac-EDDI-NH_2_ was the most potent NAA80 bisubstrate inhibitor with an IC_50_ of 0.12 μM. This inhibitor should be useful in further *in vivo* studies by micro injection in *Danio rerio* and for cell-based studies by micro injection into human cells to elucidate NAA80 function and the effects upon Nt-acetylation of actin.

## Data Availability

The original contributions presented in the study are included in the article/Supplementary Materials, further inquiries can be directed to the corresponding authors.
