# Supplementary material for: Optimized bisubstrate inhibitors for the actin N-terminal acetyltransferase NAA80
Source: Front Chem. 2023 Jun 20;11:1202501. doi: 10.3389/fchem.2023.1202501 (PMC10318143; doi:10.3389/fchem.2023.1202501)
Supplement: Supplementary file 1 [file Table1.DOCX]

Optimized bisubstrate inhibitors for the actin N-terminal acetyltransferase NAA80

Line M. Myklebust, Markus Baumann, Svein I. Støve, Håvard Foyn,
Thomas Arnesen^*^, Bengt Erik Haug^*^

| **Table S1.** Characterization of bisubstrate inhibitors by ^1^H-NMR (850 MHz, H_2_O/D_2_O, 9:1) | | | | | | |
| --- | --- | --- | --- | --- | --- | --- |
| Inhibitor | NH | Hα | Hβ | Hγ | Hδ | CONH_2_ |
| **CoA-Ac-DDEI-NH_2_^⧧^** |  |  |  |  |  |  |
| Asp^1^ | 8.71 | 4.73 | 2.96, 2.87 |  |  |  |
| Asp^2^ | 8.56 | 4.71 | 2.93, 2.87 |  |  |  |
| Glu^3^ | 8.23 | 4.39 | 2.13, 1.99 | 2.45 |  |  |
| Ile^4^ | 8.04 | 4.13 | 1.85 | 1.47, 1.18, 0.92 | 0.86 | 7.62, 7.10 |
| **CoA-Ac-DEEL-NH_2_^€^** |  |  |  |  |  |  |
| Asp^1^ | 8.72 | 4.72 | 2.96, 2.86 |  |  |  |
| Glu^2^ | 8.50 | 4.35 | 2.13, 1.98 | 2.47 |  |  |
| Glu^3^ | 8.34 | 4.36 | 2.12, 2.01 | 2.47 |  |  |
| Leu^4^ | 8.16 | 4.30 | 1.67, 1.59 | 1.61 | 0.92, 0.86 | 7.53, 7.06 |
| **CoA-Ac-DEEI-NH_2_^§^** |  |  |  |  |  |  |
| Asp^1^ | 8.71 | 4.72 | 2.95, 2.85 |  |  |  |
| Glu^2^ | 8.46 | 4.37 | 2.13, 1.97 | 2.47 |  |  |
| Glu^3^ | 8.34 | 4.39 | 2.11, 2.00 | 2.46 |  |  |
| Ile^4^ | 8.09 | 4.13 | 1.85 | 1.47, 1.18, 0.92 | 0.86 | 7.64, 7.10 |
| **CoA-Ac-EEEL-NH_2_^¶^** |  |  |  |  |  |  |
| Glu^1^ | 8.57 | 4.35 | 2.12, 2.00 | 2.49 |  |  |
| Glu^2^ | 8.50 | 4.35 | 2.09, 1.99 | 2.47 |  |  |
| Glu^3^ | 8.39 | 4.38 | 2.12, 2.00 | 2.47 |  |  |
| Leu^4^ | 8.24 | 4.31 | 1.68, 1.58 | 1.63 | 0.92, 0.86 | 7.54, 7.06 |
| **CoA-Ac-EDEI-NH_2_^√^** |  |  |  |  |  |  |
| Glu^1^ | 8.59 | 4.35 | 2.12, 2.01 | 2.48 |  |  |
| Asp^2^ | 8.61 | 4.69 | 2.94, 2.85 |  |  |  |
| Glu^3^ | 8.28 | 4.40 | 2.13, 1.98 | 2.45 |  |  |
| Ile^4^ | 8.07 | 4.13 | 1.85 | 1.47, 1.18, 0.92 | 0.86 | 7.63, 7.10 |
| **CoA-Ac-EDEL-NH_2_^🛈^** |  |  |  |  |  |  |
| Glu^1^ | 8.59 | 4.34 | 2.12, 2.01 | 2.48 |  |  |
| Asp^2^ | 8.62 | 4.68 | 2.93, 2.84 |  |  |  |
| Glu^3^ | 8.32 | 4.37 | 2.15, 1.99 | 2.46 |  |  |
| Leu^4^ | 8.15 | 4.30 | 1.69, 1.59 | 1.63 | 0.92, 0.86 | 7.49, 7.06 |
| **CoA-Ac-EEDI-NH_2_^¥^** |  |  |  |  |  |  |
| Glu^1^ | 8.57 | 4.36 | 2.12, 1.99 | 2.49 |  |  |
| Glu^2^ | 8.49 | 4.37 | 2.10, 1.97 | 2.47 |  |  |
| Asp^3^ | 8.53 | 4.74 | 2.96, 2.83 |  |  |  |
| Ile^4^ | 8.03 | 4.15 | 1.88 | 1.45, 1.17, 0.91 | 0.86 | 7.61, 7.11 |
| **CoA-Ac-EEDL-NH_2_^$^** |  |  |  |  |  |  |
| Glu^1^ | 8.57 | 4.35 | 2.12, 2.00 | 2.49 |  |  |
| Glu^2^ | 8.51 | 4.35 | 2.09, 1.97 | 2.46 |  |  |
| Asp^3^ | 8.52 | 4.71 | 2.97, 2.84 |  |  |  |
| Leu^4^ | 8.23 | 4.30 | 1.68, 1.60 | 1.60 | 0.91, 0.84 | 7.50, 7.07 |
|  |  |  |  |  |  |  |
| *Table S1. continued* | | | | | | |
| Inhibitor | NH | Hα | Hβ | Hγ | Hδ | CONH_2_ |
| **CoA-Ac-EDDI-NH_2_^*^** |  |  |  |  |  |  |
| Glu^1^ | 8.57 | 4.35 | 2.11, 1.99 | 2.48 |  |  |
| Asp^2^ | 8.59 | 4.71 | 2.94, 2.84 |  |  |  |
| Asp^3^ | 8.41 | 4.74 | 2.94, 2.84 |  |  |  |
| Ile^4^ | 7.95 | 4.14 | 1.88 | 1.45, 1.17, 0.91 | 0.86 | 7.60, 7.11 |
| **CoA-Ac-ESEL-NH_2_^¢^** |  |  |  |  |  |  |
| Glu^1^ | 8.61 | 4.38 | 2.14, 2.02 | 2.50 |  |  |
| Ser^2^ | 8.46 | 4.41 | 3.88, 3.85 |  |  |  |
| Glu^3^ | 8.39 | 4.40 | 2.16, 2.00 | 2.48 |  |  |
| Leu^4^ | 8.14 | 4.29 | 1.66, 1.58 | 1.63 | 0.92, 0.86 | 7.49, 7.06 |
| **CoA-Ac-EDQL-NH_2_^£^** |  |  |  |  |  |  |
| Glu^1^ | 8.60 | 4.35 | 2.11, 1.99 | 2.48 |  |  |
| Asp^2^ | 8.63 | 4.68 | 2.94, 2.85 |  |  |  |
| Gln^3^ | 8.40 | 4.32 | 2.13, 1.98 | 2.35 |  |  |
| Leu^4^ | 8.18 | 4.30 | 1.68, 1.58 | 1.62 | 0.92, 0.86 | 7.51, 7.06 |
| **CoA-Ac-PDEL-NH_2_^^** |  |  |  |  |  |  |
| Pro^1^ |  | 4.39 | 2.30, 1.93 | 2.01 | 3.69 |  |
| Asp^2^ | 8.57 | 4.69 | 2.95, 2.86 |  |  |  |
| Glu^3^ | 8.25 | 4.38 | 2.15, 2.01 | 2.46 |  |  |
| Leu^4^ | 8.14 | 4.30 | 1.68, 1.59 | 1.62 | 0.92, 0.86 | 7.49, 7.06 |

^⧧^CoA: *δ* = 8.69 (H8), 8.45 (H2), 8.17 (N^7’’^H), 8.06 (N^4’’^H), 6.23 (H1’), 4.90 (H2’), 4.88 (H3’), 4.61 (H4’), 4.27 (H5’), 4.04 (H3’’), 3.86 (H1’’), 3.61 (H1’’), 3.49 (H5’’), 3.37 (H8’’), 2.69 (H9’’), 2.48 (H6’’), 0.95 (H10’’), 0.82 (H10’’); Ac: *δ* = 3.33.

^€^CoA: *δ* = 8.69 (H8), 8.45 (H2), 8.17 (N^7’’^H), 8.06 (N^4’’^H), 6.23 (H1’), 4.90 (H2’), 4.87 (H3’), 4.61 (H4’), 4.26 (H5’), 4.04 (H3’’), 3.86 (H1’’), 3.60 (H1’’), 3.49 (H5’’), 3.37 (H8’’), 2.69 (H9’’), 2.48 (H6’’), 0.94 (H10’’), 0.82 (H10’’); Ac: *δ* = 3.34.

^§^CoA: *δ* = 8.69 (H8), 8.45 (H2), 8.17 (N^7’’^H), 8.06 (N^4’’^H), 6.23 (H1’), 4.90 (H2’), 4.87 (H3’), 4.61 (H4’), 4.26 (H5’), 4.04 (H3’’), 3.86 (H1’’), 3.60 (H1’’), 3.49 (H5’’), 3.37 (H8’’), 2.69 (H9’’), 2.48 (H6’’), 0.94 (H10’’), 0.82 (H10’’); Ac: *δ* = 3.34.

^¶^CoA: *δ* = 8.69 (H8), 8.45 (H2), 8.18 (N^7’’^H), 8.06 (N^4’’^H), 6.23 (H1’), 4.90 (H2’), 4.88 (H3’), 4.61 (H4’), 4.26 (H5’), 4.04 (H3’’), 3.86 (H1’’), 3.60 (H1’’), 3.49 (H5’’), 3.38 (H8’’), 2.71 (H9’’), 2.48 (H6’’), 0.95 (H10’’), 0.82 (H10’’); Ac: *δ* = 3.34.

^√^CoA: *δ* = 8.69 (H8), 8.45 (H2), 8.18 (N^7’’^H), 8.06 (N^4’’^H), 6.23 (H1’), 4.90 (H2’), 4.88 (H3’), 4.61 (H4’), 4.26 (H5’), 4.04 (H3’’), 3.86 (H1’’), 3.60 (H1’’), 3.49 (H5’’), 3.38 (H8’’), 2.71 (H9’’), 2.48 (H6’’), 0.95 (H10’’), 0.82 (H10’’); Ac: *δ* = 3.33.

^🛈^CoA: *δ* = 8.69 (H8), 8.44 (H2), 8.18 (N^7’’^H), 8.06 (N^4’’^H), 6.23 (H1’), 4.90 (H2’), 4.87 (H3’), 4.61 (H4’), 4.26 (H5’), 4.04 (H3’’), 3.86 (H1’’), 3.59 (H1’’), 3.48 (H5’’), 3.38 (H8’’), 2.71 (H9’’), 2.48 (H6’’), 0.94 (H10’’), 0.81 (H10’’); Ac: *δ* = 3.34.

^¥^CoA: *δ* = 8.69 (H8), 8.45 (H2), 8.18 (N^7’’^H), 8.06 (N^4’’^H), 6.23 (H1’), 4.90 (H2’), 4.87 (H3’), 4.61 (H4’), 4.26 (H5’), 4.04 (H3’’), 3.86 (H1’’), 3.60 (H1’’), 3.49 (H5’’), 3.38 (H8’’), 2.71 (H9’’), 2.48 (H6’’), 0.95 (H10’’), 0.82 (H10’’); Ac: *δ* = 3.33.

^$^CoA: *δ* = 8.69 (H8), 8.45 (H2), 8.18 (N^7’’^H), 8.06 (N^4’’^H), 6.23 (H1’), 4.90 (H2’), 4.87 (H3’), 4.61 (H4’), 4.26 (H5’), 4.05 (H3’’), 3.86 (H1’’), 3.60 (H1’’), 3.49 (H5’’), 3.38 (H8’’), 2.71 (H9’’), 2.48 (H6’’), 0.94 (H10’’), 0.82 (H10’’); Ac: *δ* = 3.34.

^*^CoA: *δ* = 8.68 (H8), 8.45 (H2), 8.18 (N^7’’^H), 8.06 (N^4’’^H), 6.23 (H1’), 4.90 (H2’), 4.88 (H3’), 4.61 (H4’), 4.27 (H5’), 4.04 (H3’’), 3.87 (H1’’), 3.61 (H1’’), 3.48 (H5’’), 3.38 (H8’’), 2.70 (H9’’), 2.48 (H6’’), 0.95 (H10’’), 0.82 (H10’’); Ac: *δ* = 3.33.

^¢^CoA: *δ* = 8.69 (H8), 8.45 (H2), 8.18 (N^7’’^H), 8.06 (N^4’’^H), 6.23 (H1’), 4.90 (H2’), 4.87 (H3’), 4.61 (H4’), 4.26 (H5’), 4.04 (H3’’), 3.86 (H1’’), 3.61 (H1’’), 3.49 (H5’’), 3.38 (H8’’), 2.71 (H9’’), 2.48 (H6’’), 0.95 (H10’’), 0.82 (H10’’); Ac: *δ* = 3.34.

^£^CoA: *δ* = 8.69 (H8), 8.45 (H2), 8.18 (N^7’’^H), 8.06 (N^4’’^H), 6.23 (H1’), 4.90 (H2’), 4.87 (H3’), 4.61 (H4’), 4.26 (H5’), 4.04 (H3’’), 3.86 (H1’’), 3.60 (H1’’), 3.49 (H5’’), 3.38 (H8’’), 2.70 (H9’’), 2.48 (H6’’), 0.95 (H10’’), 0.82 (H10’’); Ac: *δ* = 3.33.

^^CoA: *δ* = 8.69 (H8), 8.45 (H2), 8.17 (N^7’’^H), 8.06 (N^4’’^H), 6.23 (H1’), 4.90 (H2’), 4.88 (H3’), 4.61 (H4’), 4.27 (H5’), 4.04 (H3’’), 3.87 (H1’’), 3.61 (H1’’), 3.49 (H5’’), 3.38 (H8’’), 2.73 (H9’’), 2.49 (H6’’), 0.95 (H10’’), 0.82 (H10’’); Ac: *δ* = 3.49.
